# Scaling up peer‐led community‐based differentiated support for adolescents living with HIV: keeping the needs of youth peer supporters in mind to sustain success

**DOI:** 10.1002/jia2.25570

**Published:** 2020-08-31

**Authors:** Sarah Bernays, Maureen Tshuma, Nicola Willis, Kudzanayi Mvududu, Adrian Chikeya, Juliet Mufuka, Frances Cowan, Webster Mavhu

**Affiliations:** ^1^ Global Health and Development London School of Hygiene and Tropical Medicine London United Kingdom; ^2^ School of Public Health University of Sydney Sydney Australia; ^3^ Centre for Sexual Health and HIV/AIDS Research (CeSHHAR) Harare Zimbabwe; ^4^ Africaid Harare Zimbabwe; ^5^ Department of International Public Health Liverpool School of Tropical Medicine Liverpool United Kingdom

**Keywords:** HIV, adolescents, differentiated service delivery, peer support, community based, health systems

## Abstract

**Introduction:**

Low rates of viral suppression among adolescents living with HIV (ALHIV) indicate that more effective support is urgently required at scale. The provision of peer support has generated considerable enthusiasm because it has the potential to ameliorate the complex social and relational challenges which underpin suboptimal adherence. Little is known about the impact on young peer supporters themselves, which is the focus of this paper.

**Methods:**

We present qualitative findings from the Zvandiri trial investigating the impact of a peer support intervention on the viral load for beneficiaries (ALHIV, aged 13 to 19 years) in Zimbabwe. The Zvandiri peer supporters aged 18 to 24 years, known as community adolescent treatment supporters (CATS), are themselves living with HIV. Individual in‐depth interviews were conducted in late 2018 with 17 CATS exploring their experiences of delivering peer support and their own support needs. Interviews were analysed iteratively using thematic analysis.

**Results:**

The CATS reported that being peer supporters improved their own adherence behaviour and contributed to an improved sense of self‐worth. The social connections between the CATS were a source of comfort and enabled them to develop skills to manage the challenging aspects of their work. Two substantial challenges were identified. First, their work may reveal their HIV status. Second, managing the emotional labour of this caring work; given how commonly the complexity of the beneficiaries’ needs mirrored the circumstances of their own difficult lives. Both challenges were ameliorated by the support the CATS provided to each other and ongoing supervision from the adult mentor. There was variation in whether they felt their roles were appropriately valued through the remuneration they received and within the health system. There was a consensus that their experience meant that they would graduate from being a CATS with transferable skills that could enhance their employability.

**Conclusions:**

Their experiences illustrate the advantages and opportunities of being a CATS. To minimize potential harms, it is vital to ensure that they feel valued in their role, which can be demonstrated by the provision of appropriate remuneration, recognition and respect, and that there is continued investment in ongoing support through ongoing training and mentoring.

## 1 INTRODUCTION

A tenacious driver of the higher rates of morbidity and mortality amongst adolescents living with HIV (ALHIV) is suboptimal adherence to HIV treatment [1, 2, 3, 4] Although the global target is to get 95% of those on treatment to be virally suppressed, combined data from East and Southern African countries show that only 45% of adolescent girls living with HIV are virologically suppressed (vs. the goal of 86% under UNAIDS 95‐95‐95 targets for 2030) [5]. This makes them more vulnerable to treatment failure.

The provision of one‐to‐one peer support at scale has generated considerable enthusiasm because it has the potential to ameliorate the complex social and relational challenges that are known to affect adolescents’ capability to engage in sustained adherence to HIV treatment [6, 7, 8, 9]. Peer support is predicated on the basis that sharing knowledge and experience can provide emotional, social and practical support [10, 11, 12]. The similarity in experiences between the adolescent beneficiaries and young peer supporters provide the opportunity for effective support. However, being responsible for helping others experiencing the same problems may also exacerbate the vulnerability of peer supporters.

Community‐based peer support models have not proven to be consistently effective for children, adolescents and young people living with HIV [13]. However, the Zvandiri trial, a cluster‐randomized trial which evaluated a theoretically informed peer‐led differentiated service delivery intervention on HIV in Zimbabwe [14, 15], found 42% lower prevalence of virological failure or death at 96 weeks among participants receiving the Zvandiri intervention than among those solely receiving standard HIV care at rural clinics [16, 17]. A detailed explanation of the intervention and trial is outlined in related publications [15, 18]. The findings from this trial add to the growing body of evidence to support the scale‐up of differentiated service delivery incorporating peer‐supporters to support adolescents’ antiretroviral treatment (ART) adherence and retention in care. With an emerging investment in peer support programmes [19], there is a clear need to address the knowledge gap that exists about the impact, value and risks of such interventions for the young peer supporters themselves. In this paper, we draw on qualitative research conducted with the peer‐supporters, known as Community Adolescent Treatment Supporters (CATS), in the Zvandiri trial to explore their experiences of delivering peer support and to identify their own support needs.

## 2 METHODS

### 2.1 Study setting

The current HIV prevalence among those aged 15 to 49 years old in Zimbabwe is 12.7% [20, 21, 22]. The trial was conducted with public clinics in two rural districts (Bindura and Shamva) in Mashonaland Central province, Zimbabwe. The trial communities are characterized by high youth unemployment, a struggling economy, persistent stigma around HIV, faith healing and a stretched healthcare system with weak mental health provision.

### 2.2 Data collection

We conducted individual in‐depth interviews between November and December 2018 with 17 of the 18 CATS, aged 19 to 24 years, who were actively working in the intervention sites (n = 6 female, n = 11 male). The one active CATS not interviewed was temporarily away from the district during the fieldwork period. There were 29 CATS who had been trained but were no longer active in the trial by the time of data collection. After the initial recruitment, a large proportion left in the early stages of the trial as they found the work did not suit them. We outline, using the information available, the reasons that the 29 CATS were no longer working in Table 1.

**Table 1:** Reasons CATS had left the trial intervention

Reason for attrition	Number of CATS
Relocated	5
Employment	2
Married	3
Pregnancy	1
Lack of engagement (competing priorities, limited time to commit to CATS’ duties, inadequate commitment)	18
Total	29

CATS, Community Adolescent Treatment Supporters.

We explored how the CATS considered that the intervention affected their lives, their experiences of being involved and their support needs. Individual interviews were conducted by one male and two female Zimbabwean researchers (AC, MT and KM) in private spaces in the public clinics and lasted approximately an hour. All the interviews were conducted in Shona, the participants’ language.

With the participants’ permission, the interviews were audio recorded. The interviewers listenned back to the recordings and wrote up the interviews into detailed interview scripts [23, 24]. Iterative data collection and analysis was conducted, in which the team discussed each interview in weekly analytical meetings. This informed the refinement of the topic guides. Our theoretical approach was informed by social constructionism with particular attention paid to relational agency, which is attentive to the restricted agency young people may have and how this is framed by their relationships, but is also fluid and so may be dynamic and transformative [25].

A thematic analytical approach was adopted, which involved SB, MT, AC and KM coding the scripts to develop a coding framework. Excel was used to organize the coding. Themes were developed through data‐led analytical memos. Selected extracts were transcribed verbatim and translated for equivalent meaning in English. SB and WM provided analytical training to MT, AC and KM throughout 2018 and 2019.

Ethical approval was granted by the Medical Research Council of Zimbabwe (#2032) and the ethics committees of the London School of Hygiene & Tropical Medicine (London, UK; #11042) and University College London (London, UK; #2358/004). Written informed consent was collected prior to participation. Pseudonyms are used for all participants.

## 3 RESULTS

### 3.1 Feeling valuable: helping them, helps me

#### 3.1.1 Changes in adherence behaviour

In general, the CATS reported that working as a CATS had brought them considerable opportunity and advantage, including improving their capacity to manage their HIV status. Although the remit of the CATS was to support other ALHIV, many of them themselves had been struggling with adhering to treatment: “Prior to being a CATS, I would take them anytime (ARVs). If I wasn’t reminded at times I would skip.” (Progress, female, aged 20). Many reported that the training and education which they received through the programme had improved their treatment literacy and galvanized their commitment to compliance: “I only got to know about all of this when I became a CATS.” (Rudo, female, aged 24).

All of the CATS who had previously struggled with adherence described how the reminder structures they put in place for beneficiaries helped them too: “I send adolescents reminders, so there is no way that I won’t also take my medication at seven o’clock.” (Geoffrey, male, aged 20); “when you see the impact of those that are not taking their medication on time and think about yourself who is taking on time you realise there is a difference.” (Taurai, male, aged 21). These insights encouraged them to comply.

“When I became a CATS and seeing others this gave me strength that even if I wanted to default I should know it’s my own life being affected. From then, it has helped me to know that medication should be taken correctly and on time.” (Rudo, female, aged 24)

Overall the CATS considered their work fulfilling and were proud that the support they offered had a positive impact. The majority considered that the proximity of their own experiences to those of the beneficiaries enabled them to make a unique contribution, which underpinned why they were so effective:

“This is work that I enjoy as l work with my age‐mates. I am able to relate to them well… it may be difficult for adults to understand them.” (Lisa, female, aged 20).

Many of them reported that they had developed an increased sense of self‐worth, which motivated their investment in their own health. Farai explained the mutual benefit of the peer support that he offered:

“I find myself really happy because of the interaction with the adolescents, giving them support and sharing with them what I have gone through. I am able to motivate them. It is really helping me too because we share problems with the adolescent on what they are going through and what I am going through. We help each other.” (Male, aged 22)

#### 3.1.2 Gaining comfort and strength from the CATS’ community

For most CATS their experience of living with HIV prior to engaging with the Zvandiri programme had been defined by solitude. They described having been burdened by anxiety about the anticipated loneliness and limited prospects that would characterize their futures. The CATS had regular and various opportunities to interact with each other through the coordination meetings and reported deriving significant benefits from engaging in a community of young people affected by HIV. Feeling accepted and connected transformed their expectations of what it meant to live with HIV: “I realised that I was letting myself down. This programme gave me confidence to love myself.” (Lazarus, male, aged 20).

Sharing their experiences was also instrumental in developing their capacity to deliver their duties as CATS. They consistently emphasized the value of being able to collectively develop support strategies and resolve problems in the regular discussions they had with the other CATS and mentors. “We help each other and sharpen each other intellectually with ideas” (Tapiwa, male, aged 20). Progress reflected a widely held opinion: “I have learnt a lot including that if you face any challenge don’t make it your own but share it with others so that you can help each other solve it.” (Female, aged 20).

### 3.2 Dilemmas in being “seen” as a CATS

The Zvandiri intervention was new to the districts in the trial. So the CATS were a new cohort and had no prior experience of the intervention. The youngest CATS to be recruited at the start of the trial were 18 years old. Many were still in the process of accepting their HIV status. Despite the internal progress made around self‐acceptance, they were mindful of prevailing stigmatizing attitudes that persisted within their local communities. Consequently, the majority of CATS concealed the nature of their work from others in the community. As Lazarus explains:

“I don’t show them what the job entails, but they try to find out what I do. I am an open person but … I am not able to do that (to talk about his status in public).” (Male, aged 20).

Public disclosure of one’s HIV status is not a requirement of being a CATS. For a significant minority the risk of their role, and consequently their HIV status, being discovered was a source of worry and stress. They described finding this difficult to manage, particularly in the first months of working as a CATS. Although the risk persisted, with support and guidance from other CATS and their mentors, their anxiety tended to ease over time as they became more robust in managing other people’s questions. Geoffrey described a common pattern in his reaction to uncomfortable questions from those within the wider community:

“This mainly happened in the first days… At first it pained me and you would really have so many different thoughts… (Fellow) CATS will then say to me ‘my friend, I came across this numerous times. They are not people to listen to, what you need to do is just ride your bicycle and go’. I ended up doing that. Now I have a good relationship with them and no one asks me what I am doing.” (Male, aged 20).

There were two exceptional cases when individuals had less control over who knew about their status within the community. For one CATS his HIV‐positive status had been widely presumed because as a child he had been known as a “sickling.” In the second case a healthcare worker had disclosed a CATS’ HIV‐positive status to other patients at the clinic. Although this has been “a painful experience,” it was attributed to a rare act of thoughtlessness by the healthcare worker. In general, the risk of deductive disclosure in which their HIV positive status was revealed by virtue of being eligible to work as a CATS appeared to be low and to be well managed by both the young people, their mentors and others involved.

### 3.3 Critical support needs to offset risks of being a CATS

Events such as falling sick, struggling with their mental health or problems within their own households were rare, but did at times influence their capacity to fulfil the obligations of the role. In addition, hearing about the shared challenges they faced as young people growing up with HIV, contributed to the CATS’ precarious vulnerability. The substantial support offered by the CATS’ mentors was a critical factor mediating the emotional burden which accompanied their work. On some occasions this involved the mentors needing to be flexible about an individual CATS’ workload.

The Zvandiri mentors, adults with professional experience caring for adolescents living with HIV, provided training, as well as ongoing supervision to the CATS and took on the management of mental health referrals and support in complex cases. The counselling and personal support provided by the mentors played a pivotal role in enabling the CATS to continue to provide support, without being overwhelmed or risk “burn‐out.” Sarudzai recalled how her mentor “follows up every week to check on me” (female, aged 20), which acted as a safety net to manage the daily stresses that could otherwise disrupt the CATS’ adherence and wellbeing. Practically, the mentors also focused on keeping the number of cases that a CATS was responsible for to fewer than ten and equipping them with bicycles so that they could cover the relatively long distances to complete the required number of home visits. These were pivotal management strategies to support the CATS in their work.

In the first few months of their role as CATS, young people had to absorb a lot of information, implement new skills and develop relationships with beneficiaries and caregivers. Concomitantly, having just finished school and entering the employment market, they were by definition very early on in their careers as caring professionals. In common with anyone embarking on a career in a caring profession, the CATS were still learning how to manage the burden of responsibility involved in their role. They praised the mentors and other CATS in supporting them through this early period. For example, the mentors commonly facilitated introductions of CATS to households where caregivers were initially reticent for their child to be engaged in the programme. Over time it became easier, but ongoing support remained critical:

“I say it's easy (CATS work) because you are not left to work with the adolescents by yourself. In most cases I work with others and you are helped if you have any questions… It’s the mentor that supports me.” (Lisa, female, aged 20)

Without exception the CATS reported feeling a keen sense of responsibility for the health and wellbeing of their beneficiaries. When beneficiaries received high viral load results it was not uncommon for the CATS to feel “hurt,” “disappointed” and “worried.” Chipo (female, aged 21) explains how in such instances, “I may blame myself and wonder if I am failing to do my work or is it the adolescent who is just not understanding.” The impact was even more acute when a beneficiary died.

In general, the opportunity to engage the mentor when they were struggling and stressed was pivotal to CATS being able to manage the related stresses of complex cases. Part of this involved acknowledging that some of the problems that beneficiaries encountered were likely to be beyond the remit of what the CATS could be expected to influence. Managing their expectations was key: not all of the problems could be resolved, but with the intervention’s support they might be ameliorated. “She was able to help with advice… well she was able to help to a certain level which made the situation lighter though this didn’t completely solve the problem.” (Chipo, female, aged 21).

Despite the pressures, the CATS were generally very positive about the consistency of the support that they received. There were only a few instances when the provision of support was considered to have been late or insufficient. The negative reactions of CATS when support was considered inadequate, although rare, further reinforces the importance of timely, responsive and proactive support to mitigate the risks of being a CATS.

### 3.4 Being valued: the role of remuneration and recognition

#### 3.4.1 Competing commitments: working in my household and working doing home visits

The CATS considered themselves to be both valuable and valued in their roles. The stipend they received for their work was welcomed by most of the CATS. Those who could generally spend it on their own needs consistently described it as helpful: “I have been able to do certain things like I managed to take my class four (driver’s licence) as well as to make a part payment for a course (operating earth moving equipment).” (Tanatswa, male, aged 20). However, many CATS had additional responsibilities and needed income to support their dependents and they were more likely to describe the remuneration as insufficient. Some had contemplated quitting to take up work with better pay.

The relatively “low” remuneration (US$20 a month) paid to the CATS created challenges in how their role was perceived within their own households, as well as within the clinics. For a minority, their limited earning was a source of household tension. The remuneration was considered inadequate to justify the time spent away from household chores.

“At home I was not being treated well … they were giving me a hard time. They would always say ‘you are running away from doing chores, the money you are getting is peanuts. How can you work for only twenty dollars (US$)?’” (Tinashe, male, aged 23).

The competing demands of contributing to their household and fulfilling their duties as a CATS was most apparent during farming seasons when the CATS often had to stay at home to work in the gardens: “I will be preoccupied with other household chores … as I will be at home some of the time because I am the head of the house.” (James, male, aged 22).

#### 3.4.2 Recognition of skills to support transitioning on

In the first few months of the intervention, healthcare workers reportedly expressed some ambivalence about the contribution that the CATS could make. However, over time most of the CATS described feeling recognized and valued within the clinics where they worked. “They (healthcare workers) say ‘you are clever, you are doing your work well’ … I start to feel elated and I know that it is really working well.” (Chipo, female, aged 20). The vast majority of the CATS articulated their hopes that the CATS’ role would be formally recognized within the health system.

Being a CATS is a time limited role, with young people unable to continue once they are 25 years old. Many were hoping that they would go on to be employed by the Zvandiri programme but recognized that this was a narrow option only available to a few. In general, the transferable skills they gained through their training and professional experience increased their employability. There were some challenges in describing their work experience because without a generic qualification, having to describe the precise nature of their role risked deductive disclosure. In some cases, CATS transitioned to taking on other roles in the clinics or benefitting directly from sponsorship opportunities:

“I collected my school results (ordinary level) recently. There are subjects I am supposed to rewrite. The nurse from this clinic has offered to pay my examination fee to supplement these subjects that I want to write. So for me, there are many opportunities.” (Farai, male, aged 22)

## 4 DISCUSSION

Increasing attention is now being paid to community‐based peer‐support programmes as a mechanism to improve clinical outcomes and wellbeing [19, 20, 21]. While the predominate focus of research on peer support for ALHIV has focused on the potential benefits to those receiving peer support [26], this study demonstrates that peer supporters themselves also benefit through improved adherence behaviours and self‐confidence. But it was characterized as emotionally and at times physically gruelling work, resonating with existing research on other peer support programmes [19, 27]. These findings illuminate the specific challenges and needs that might arise for young peer supporters providing HIV support in resource‐stretched settings. Despite the celebration of the effects of peer support, the discursive emphasis on the “adolescent” and/or “youth” characteristic of the CATS may act as a glass ceiling, inadvertently justifying not adequately valuing the contribution that they make in their title or in their pay.

There needs to be a formalized recognition of their contribution within the health system. There is currently some tension in how they are seen. This could be overcome if they were described as paid lay counsellors, rather than volunteers who receive a stipend. The CATS are not acting in a peer educator or navigator role but operating as skilled peer counsellors. Equivalent adult cadres, ordinarily working in the role of primary counsellors, are remunerated more than they are; CATS currently receive marginally more than village health workers [28]. While the CATS entry qualifications may be less and their initial training shorter than the standard six‐month duration for primary counsellors, the CATS as young adults receive training and supervision through the work that they are doing. To accelerate their integration, more emphasis needs to be placed on the initial preparatory work with healthcare workers in the clinic and caregivers within the households to assuage initial resistance. Given that many CATS described the resistance they encountered as dispiriting, this may be an opportunity to mitigate early attrition.

If there were more formal recognition of CATS as primary lay counsellors, for example where they graduated with a certified accreditation, this would also support their ability to demonstrate their impressive skillset once they transition on from being a CATS. This would avoid the current deductive disclosure risks that accompany explaining their prior role as a CATS. Instead, it would equip them with neutral language to describe the skillset and experience that they have gained, enabling them to leverage the employment opportunities that their experience warrants; a need recognized but rarely actioned in HIV youth activities more generally [29].

Although many of the CATS’ support needs relate to their age and shared HIV experiences, they also reflect those that would be required by anyone, regardless of age or HIV status, on their initiation into a professional caring role. To support the implementation of the intervention at scale, continuing to budget for intensive mentor support for new cohorts of CATS and investing in it as an integral principle of youth peer support is vital [26]. This would ensure that there is adequate support to ameliorate the cluster of challenges relating to stakeholder resistance, concerns around the occupational risks of deductive disclosure and related stigma [30, 31] and under‐appreciating the value of peer‐supporters, which can occur upon initial roll out of the intervention within communities. The findings demonstrate the supportive value that the CATS gain from each other, in common with various manifestations of peer support [32], but also illustrate that this can be further reinforced by the opportunities to mentor and be mentored through the provision of ongoing training and coordination meetings with peers [17].

A strength of this study was attending to the experiences of those providing peer support, making a valuable contribution to the emerging evidence‐base that has generally focused narrowly on the recipients. However, by not interviewing those who had already left the programme, the results may be biased towards those with more positive experiences as peer supporters. Further research with those who work only briefly as peer supporters is required to understand the factors shaping attrition. However, a pertinent distinction should be drawn between peer supporters affected by attrition, who stop due to the effects of inadequate pay, support or poor health and *transition*, when individuals move onto other opportunities reflecting their successful growth in navigating considered decisions about becoming parents or seeking more sustainable employment opportunities. From the available information among the 29 inactive CATS, the latter was more common. Without further research there is a risk that attrition rates may be over‐estimated. Peer supporters who move on before they exceed the age threshold may not represent an intervention failing, but instead indicate successful “graduation”.

## 5 CONCLUSIONS

The experiences of the CATS demonstrate the considerable personal value that they gain from their work. To be able to maximize the benefits and minimize potential occupation harms over time, ongoing intensive and age‐appropriate training and mentoring, as well as the establishment and maintenance of systems and remuneration which values the peer support role that CATS play, are integral to the success of peer support.

## COMPETING INTERESTS

Authors declare no competing interests.

## AUTHOR’S CONTRIBUTIONS

SB was the lead social scientist, designing the process evaluation study with WM and FC which forms the basis of the data presented in this manuscript. MT, KM and AC collected the data with the support of SB, WM, NW and JM. All authors contributed to the overview of data collection and preliminary analysis for this paper through focused team discussions. SB, MT, KM, AC and WM conducted the data analysis. SB developed the manuscript with support from WM, MT, KM and AC. All authors read, reviewed and approved the final manuscript.
